# Expression of Endogenous Putative TSH Binding Protein in Orbit

**DOI:** 10.3390/cimb43030126

**Published:** 2021-10-27

**Authors:** Mohd Shazli Draman, Fiona Grennan-Jones, Peter Taylor, Ilaria Muller, Sam Evans, Anjana Haridas, Daniel S. Morris, D. Aled Rees, Carol Lane, Colin Dayan, Lei Zhang, Marian Ludgate

**Affiliations:** 1 School of Medicine, Cardiff University, Heath Park, Cardiff CF14 4XN, UK; shazlidraman@gmail.com (M.S.D.); grennanjonesfa@icloud.com (F.G.-J.); taylorpn@cardiff.ac.uk (P.T.); ilaria.muller@unimi.it (I.M.); ReesDA@cardiff.ac.uk (D.A.R.); DayanCM@cardiff.ac.uk (C.D.); Ludgate@cardiff.ac.uk (M.L.); 2 KPJ Healthcare University College, Kota Seriemas, Nilai 71800, Malaysia; 3 Department of Clinical Sciences and Community Health, University of Milan, 20122 Milan, Italy; 4 Department of Endocrinology, Fondazione IRCCS Ca’ Granda Ospedale Maggiore Policlinico, 28, 20122 Milan, Italy; 5 Department of Ophthalmology, Cardiff & Vale University Health Board, Cardiff CF14 4XW, UK; samboevans@mac.com (S.E.); anjana@doctors.org.uk (A.H.); dsm@doctors.org.uk (D.S.M.); carollanedm@gmail.com (C.L.)

**Keywords:** Graves’ Orbitopathy, thyrotropin receptor, variant, binding protein, thyrostimulin

## Abstract

Thyroid stimulating antibodies (TSAB) cause Graves’ disease and contribute to Graves’ Orbitopathy (GO) pathogenesis. We hypothesise that the presence of TSH binding proteins (truncated *TSHR* variants (*TSHRv*)) and/or nonclassical ligands such as *thyrostimulin* (*α2β5*) might provide a mechanism to protect against or exacerbate GO. We analysed primary *human* orbital preadipocyte-fibroblasts (OF) from GO patients and people free of GO (non-GO). Transcript (QPCR) and protein (western blot) expression levels of *TSHRv* were measured through an adipogenesis differentiation process. Cyclic-AMP production by TSHR activation was studied using luciferase-reporter and RIA assays. After differentiation, *TSHRv* levels in OF from GO were significantly higher than non-GO (*p* = 0.039), and confirmed in ex vivo analysis of orbital adipose samples. TSHRv western blot revealed a positive signal at 46 kDa in cell lysates and culture media (CM) from non-GO and GO-OF. Cyclic-AMP decreased from basal levels when OF were stimulated with TSH or Monoclonal TSAB (M22) before differentiation protocol, but increased in differentiated cells, and was inversely correlated with the *TSHRv*:*TSHR* ratio (Spearman correlation: TSH r = −0.55, *p* = 0.23, M22 r = 0.87, *p* = 0.03). In the bioassay, TSH/M22 induced luciferase-light was lower in CM from differentiated GO-OF than non-GO, suggesting that secreted TSHRv had neutralised their effects. *α2* transcripts were present but reduced during adipogenesis (*p* < 0.005) with no difference observed between non-GO and GO. *β5* transcripts were at the limit of detection. Our work demonstrated that *TSHRv* transcripts are expressed as protein, are more abundant in GO than non-GO OF and have the capacity to regulate signalling via the *TSHR*.

## 1. Introduction

Graves’ Orbitopathy (GO) is a frequent clinical manifestation of Graves’ disease (GD) with around 30–50% of patients being affected [1,2,3,4]. Rarely the GO is severe enough to lead to blindness. There is a close association between GO and GD, implying an autoimmune response to common antigen/s in the thyroid gland and orbit. Since the *TSHR* is found to be expressed in orbital fat [5,6,7,8], and the majority of hyperthyroid patients with GO have thyroid stimulating antibodies (TSAB), the receptor is the likely antigen candidate [9]. There is female preponderance towards GO, with a 6:1 female-to-male ratio. An increase in adipogenesis and hyaluronan production produces disfiguring exophthalmos and explains the signs and symptoms of GO. Furthermore, most patients with GO have poor quality of life [10] and suffer perpetual psychological distress due to the disfiguring appearance of the exophthalmos [11]. Current managements for GO are sub-optimal, and further research is needed to understand the pathophysiology of the condition. This will lead to earlier diagnosis, thereby promoting preventative interventions and improving long-term morbidity and socioeconomic impact.

We and others have demonstrated that activation of the thyrotropin receptor (*TSHR*) in orbital preadipocyte-fibroblasts (OF) leads to increase in adipogenesis and hyaluronan production [12,13]. During adipogenesis, *TSHR* expression has been shown to increase [5] but little is known about the effects of *TSHR* activation at various differentiation stages. Thyrostimulin, a nonclassical ligand for the *TSHR* has been described, it comprises α2 and β5 subunits [14]. It is suggested that it works via paracrine effects as it has not been detected in the circulation as supported by the recent work of the Williams group in bone [15]. Over-expression of α2 subunit in transgenic mice had no overt GO phenotype but overexpression of β5 caused hyperthyroidism, weight loss and more importantly exophthalmos [16]. These facts suggest it may have a role in GO pathogenesis.

Multiple *TSHR* variants (*TSHRv*) have been described. They lack the transmembrane domain and if expressed as soluble receptor could serve as binding proteins to TSH, TSAB and thyrostimulin. Their transcripts have been detected in OF and the thyroid gland [17]. Of interest, the exon 1–8 variant demonstrated similar structure to the *TSHR* A-subunit which is generated following cleavage of the full length receptor [18,19]. Furthermore, immunisation with *TSHR* A-subunit is more effective in murine models of GD and GO than with the complete *TSHR* [20,21]. Thus these variants could have a role in GO pathogenesis by inducing further production of TSAB or protect against GO by either inducing immune reaction or ‘neutralising’ TSAB, respectively.

We have investigated the possible influence of *thyrostimulin* and *TSHRv* using in vitro cell model and ex vivo analysis of orbital adipose tissues derived from people with GO and non-GO controls.

## 2. Materials and Methods

All reagents were obtained from Sigma-Aldrich (Dorset, UK) and tissue culture components from Cambrex (Thermo Fisher Scientific, Waltham, MA, USA) unless otherwise stated.

### 2.1. Cells and Tissues Studied; In Vitro Culture and Ex Vivo Samples

*Human* Adipose Tissue was collected with informed written consent and approved by the South East Wales Research Ethics Committee (30 May 2006) with registration number (06/WSE03/37). Samples for in vitro studies were from five GO patients (two males and three females), median age of 50 years (range 39–54 years) who were diagnosed on clinical grounds based on the presence of typical clinical features and positive *TSHR* antibody. The GO samples were obtained from patients with inactive disease (Clinical activity score < 2) undergoing decompression surgery. Only one patient had had steroid therapy and was on the treatment during orbital decompression. None of these patients had previous orbital radiotherapy. The non-GO control samples (*n* = 5; three males and two females, median age 53 years old (range 52–60 years) were from individuals free of thyroid or other inflammatory eye disease who underwent eye lid surgery for cosmetic reasons. OF cells for study were obtained from explant cultures as previously described [12]. Briefly, orbital fat biopsies were diced and placed in six well plates in complete medium (CM, Hams F12, Dulbecco’s Modified Eagle Medium, 10% foetal calf serum, Penicillin/Streptomycin, pyruvate and bicarbonate) and allowed to attach so that OF migrated out from the tissue. Once OFs were adherent, the plates were washed with culture media and OFs were grown to confluence. The CM was replaced every three days. The cells were trypsinised and frozen in liquid nitrogen until further use. Cells were used at low passage number (≤ 3) thus not every sample was used for each experiment.

Samples for ex vivo analysis comprised intact samples of orbital adipose tissues from nine GO patients (three males and six females), median age of 45 years (range 32–71 years) and five non-GO controls (All females), having a median age of 66 years (range 52–86 years). The tissue was snap frozen in liquid nitrogen directly following surgical removal. Prior to RNA extraction they were pulverised in liquid nitrogen and then processed as described below.

### 2.2. In Vitro Adipogenesis

The various cell populations were plated in six well plates in CM. Adipogenesis was induced in confluent cells by replacing with differentiation medium (DM) containing 10% FCS, biotin (33 µM), pantothenate (17 µM), T3 (1 nM), dexamethasone (100 nM), thiazolidinedione (1 µM), and insulin (500 nM) for 15 days. Adipogenesis was quantified by measuring transcripts for terminal markers of differentiation lipoprotein lipase (*LPL*) by quantitative PCR (Q-PCR).

### 2.3. QPCR Analysis

RNA was extracted using Tri Reagent (Sigma) from the OF and ex vivo samples using RNA easy lipid tissue mini kit (Qiagen) according to the manufacturer’s instructions. One µg RNA was reverse transcribed using oligodT and M-MLV reverse transcriptase (Promega). cDNA was PCR amplified using primers in Table 1 for *TSHR*, *TSHR* variants and *thyrostimulin*. *TSHR* (human NM_000369) could be distinguished from *TSHRv* isoforms (human NM_001018036) by using exon 9 and 10 primers that are not present in the truncated isoforms and using unique sequence in intron 8 of the isoforms that are not present in full length *TSHR*. Sequencing of the amplicon produced confirmed the specificity of the primers for the different types of *TSHR*. Q-PCR was carried out using Brilliant II SYBR green master mix (Agilent Technologies, Stockport, UK) and a Stratagene (La Jolla, CA, USA) MX3000 light cycler. Levels were normalised against adenosine phosphatylribosome transferase (*APRT*). Standard curves were created using the relevant PCR product cloned into pGEM T-easy (Promega, Southampton, UK) except for *TSHRv* (serial dilutions of quantified PCR amplicon were used) to calculate the copy number/1000 *APRT* copies (*APRT* expression has been previously shown to be unaffected by differentiation). In a single Q-PCR experiment, all measurements were made in duplicate.

### 2.4. Production of Antibody Specific for TSHRv

We used the 21 amino acid sequence derived from intron 8 of the TSHR, which is unique to the 1.3kb TSHRv to generate a polyclonal antibody using a commercial company (Generon, Slough, UK). A synthetic peptide was produced and coupled to keyhole limpet haemocyanin and then used to immunise two *rabbits*. The resulting polyclonal antibodies were affinity purified on the immunising peptide. We verified the specificity of each antibody using western blots (described below) on the immunising peptide.

### 2.5. Western Blot Analysis

Cell lysates from OF at various time points before and during adipogenesis were obtained by addition of lysis buffer as previously described [13]. The culture supernatants from the same time points were also collected and concentrated using spin columns (Merck Millipore, Watford, Hertfordshire, UK) to produce an 80-fold concentration. Lysates and concentrated supernatants were separated using SDS-PAGE as previously described [13]. Briefly, proteins were extracted, at various time points, in Laemmli buffer containing 1 mM phenylmethylsulphonyl fluoride. Samples were separated by 10% SDS-PAGE and then the gel electroblotted onto PVDF membrane. The blots were probed using antibodies to the full length TSHR (2C11, Santa Cruz Biotechnology, Heidelberg, Germany) and TSHRv antibody, at dilutions of 1:200 and 1:50 respectively at 4 °C overnight. This was followed by a *sheep* anti-mouse IgG-HRP (1:5000, room temperature for 1 h, GE Healthcare) or *donkey* anti-*rabbit* IgG-HRP conjugate (1:5000, room temperature for 1 h, GE Healthcare) and then visualised by enhanced chemiluminescence using ECL Plus (Amersham Pharmacia Biotech, Buckinghamshire, UK). They were then stripped and reprobed with antibodies to housekeeping protein, actin at dilution of 1:1000 4 °C overnight with secondary anti-rabbit as above.

### 2.6. Measurement of TSHR Activation

*TSHR* activation was measured in two different contexts. In the direct assay, OF were cultured in 12 well plates at various time points before and during adipogenesis. Prior to the assay they were switched to serum free medium for 24 h and then treated with IBMX 10^−4^ M alone or combined with bovine TSH 5 mu/mL or monoclonal TSAB (M22, RSR) 0.2 ng/µL 37 °C for 4 h. The cells were then lysed using HCl (0.1 M) and the cAMP in the lysate measured using an in-house radio-immunoassay, as previously described [22].

In the indirect assay, culture supernatants from OF before and at various time points during adipogenesis were collected as described above. The supernatants were then added to TSH or M22 whilst they were assayed using an in-house luciferase bioassay as previously described [23]. Briefly, 2 × 10^4^ lulu (CHO cells expressing the full-length *human TSHR* and a cAMP-responsive luciferase reporter) and zulu (CHO cells expressing the cAMP-responsive luciferase reporter but not the *TSHR*) were plated in triplicates in 96 well plates in complete medium for 48 h prior to assay. On the day of assay, the cells were washed with PBS and culture supernatants added with or without (control) TSH or M22. This was incubated at 37 °C for 5 h prior to addition of passive lysis buffer (Promega) and frozen at −80 °C prior to analysis. The analysis was performed with luciferase assay reagent (Promega, Madison, WI, USA) using Glomax Multidetection System (Promega). Stimulation index was calculated by dividing mean of lulu* light unit for each sample by the mean of zulu light unit.

### 2.7. Statistical Analysis

For statistical analysis, we used SPSS 18.0 software. Where appropriate, data were analysed using Student’s *t* test for parametric and Mann-Whitney for nonparametric. A paired t-test and One-way ANOVA with post-hoc Tukey were carried out where indicated. In all cases, *p* < 0.05 was considered significant. The statistical analysis applied is indicated in the tables and figure legends. All parametric data are presented as mean ± sem and median ± interquartile range for nonparametric.

## 3. Results

### 3.1. TSHRv Transcripts Are Expressed at Higher Levels than Full-Length TSHR

Adipogenesis in OF was induced in about 10% of cells and, as in previous reports [24], we observed that GO OF cells had higher adipogenic capacity than those from non-GO patients (Supplementary Figure S1).

**Figure 1:**
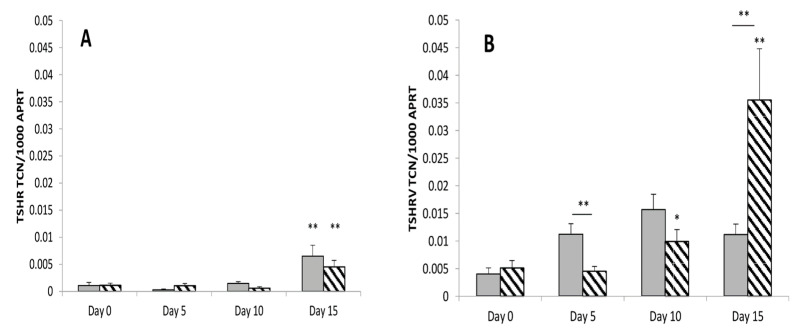
The full length *TSHR* (**A**) and the *TSHRv* (**B**) transcripts were measured in GO (Stippled bar, *n* = 5) and non-GO OF (Grey bar, *n* = 5) at multiple time points before (Day 0) and during adipogenesis (Day 5, 10, 15). Results are expressed as transcript copy number of *TSHR* or *TSHRv* per 1000 copies of the housekeeper *APRT*. Results are expressed as mean ± SEM; * *p*-value < 0.05, ** < 0.01 compared to day 0 on each respective day. Horizontal bars represent comparison between non-GO and GO.

During adipogenesis, the full-length *TSHR* expression increased. There was no significant difference between GO and non-GO full-length *TSHR* expression as shown in Figure 1A. The *TSHRv* transcripts were expressed at higher levels than the complete receptor and its level increased during differentiation, as shown in Figure 1B. Furthermore, the *TSHRv* transcripts were expressed at significantly higher levels in GO than non-GO, possibly reflecting the higher adipogenic potential of these cells. As a result, the ratio of *TSHRv* to *TSHR* varied between 5 and 25 but was highly variable, so that no significant difference was observed in the ratio between cultures from GO and non-GO.

### 3.2. There Is No Role of Thyrostimulin in GO

*Thyrostimulin α2* transcripts were detected in OF. There was a reduction in transcript levels during adipogenesis (*p* < 0.05) (Supplementary Figure S2) with no difference was found between non-GO and GO OF. In contrast *β5* transcripts were not detected.

### 3.3. Transcripts Expression of Thyrostimulin and TSHRv in Ex Vivo Samples

Ex vivo analysis were performed on orbital adipose tissues after surgical removal. *TSHRv* transcripts were detected with no differential effect between non-GO and GO. Similar to in vitro cultures’ finding, the *TSHRv* transcripts were more abundant than full length *TSHR* (Supplementary Figure S3) and the *TSHRv*:*TSHR* ratio was no different. We confirmed the findings of in vitro cultures that *α2* transcripts of thyrostimulin were detected in the ex vivo samples while *β5* transcripts were undetectable and there was no difference between GO and non-GO.

### 3.4. TSHRv and TSHR Protein Are Detectable in OF

**Figure 2:**
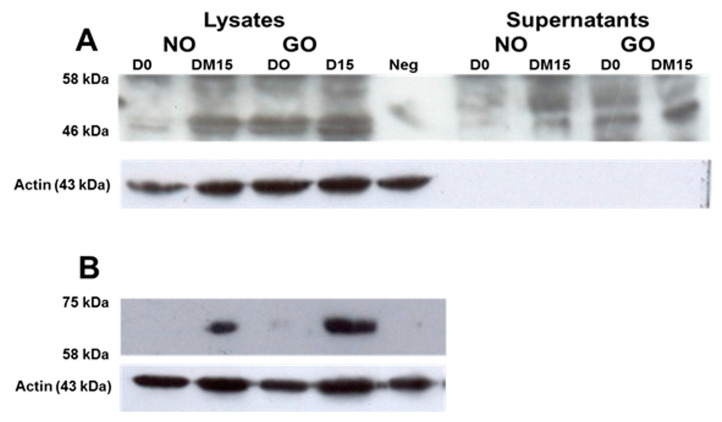
Western blot analysis of OF lysates and supernatants at baseline (D0) and after adipogenesis (DM15) from a GO patient (GO) and non-GO (NO); (**A**) antibody to TSHRv (**B**) 2C11 antibody to full-lengthTSHR. Neg; negative control (Secondary antibody only).

We generated an antibody specific for the TSHRv (Supplementary Figure S4). We then performed western blots analyses on concentrated culture supernatants and cell lysates a from non-GO and GO OF. We detected a protein with an apparent molecular mass of 46 kD in the cell lysates and culture supernatants from OF (Figure 2A). This is consistent with the TSHRv, which consists of 253 amino acids and retains five of the six putative N-linked glycosylation sites present in the full-length TSHR. The doublet signals may represent differing amounts of protein glycosylation. Its presence in the conditioned culture medium suggests that it can be secreted from cells.

Similar to the transcript findings, the protein levels increased in samples prior to and after adipogenesis. There was a stronger signal in the GO samples compared with non-GO, both in the cell lysates and conditioned culture medium. Using 2C11 antibody (which detects full length TSHR), we also demonstrated that the full length TSHR protein with an apparent molecular mass of 62kD was present in the cell lysates after adipogenesis and higher in GO than non-GO Figure 2B (*n* = 3).

### 3.5. Is the TSHRv Functional?

cAMP productions were measured in response to a *human* monoclonal TSAB (M22) and TSH at multiple time points before (basal, day 0) and after differentiation (day 15) of OF. At basal levels, we noted a decrease in cAMP production when OF were stimulated with M22 or TSH in GO compared to no-change in non-GO (*p*-value < 0.05). The opposite was noted in differentiated cells, where there was an increase in cAMP following M22 or TSH treatment, as shown in Table 2. The difference between GO and non-GO was not significant.

**Figure 3:**
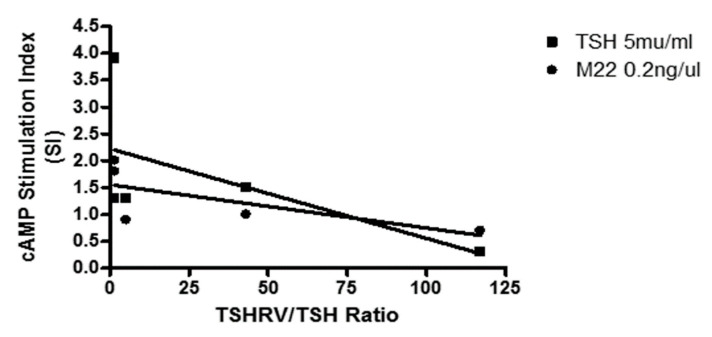
cAMP stimulation index (SI) was negatively associated with *TSHRv*:*TSHR* transcripts ratio.

Through adipogenesis, the amount of cAMP generated was negatively associated with *TSHRv*:*TSHR* transcripts ratio (Figure 3) but this achieved significance only for M22 (Spearman correlation: TSH r = −0.55, *p* = 0.23, M22 r = 0.87, *p* = 0.03).

The ability of conditioned medium from OF’s before and after differentiation to interfere with M22 or TSH induced TSHR activation was assessed in a luminescent bioassay. Standard assay was performed in serum-free medium and both M22 and TSH induced a dose-dependent stimulation index (S.I. as detailed in Method section) as shown in Supplementary Figure S5.

**Figure 4:**
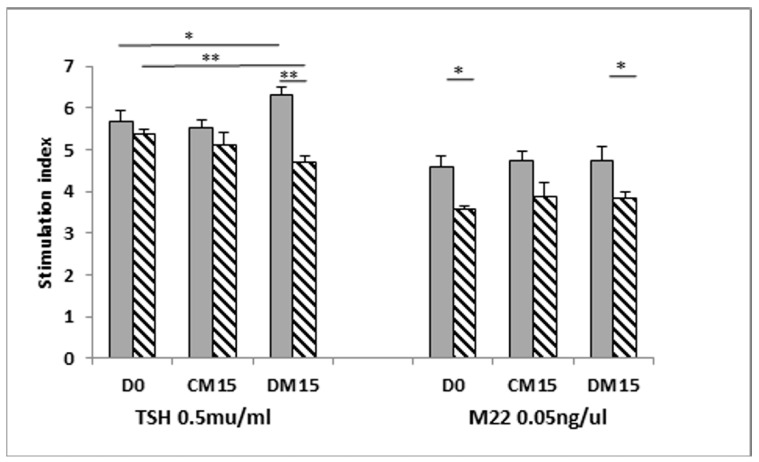
Luminescent bioassay to measure cAMP following TSHR activation. Indirect assays to determine whether the TSHRv is functional were conducted using OF conditioned medium at various stages of differentiation (cells at D0 = Day 0 in complete medium, CM15 = complete medium for 15 days culture, DM15 = Differentiation medium for 15 days) in the presence of TSH/M22. Results are expressed as mean ± SEM; * *p*-value < 0.05, ** *p*-value < 0.01. Horizontal bars represent comparison between non-GO (grey) and GO (striped).

We then used basal (day 0) conditioned medium (serum-free) from confluent cells prior to addition of differentiation medium as the baseline S.I. There was no significant difference in TSH-induced S.I. between non-GO and GO conditioned medium from undifferentiated cells at day 0 (D0) and day 15 (CM15). At the end of adipogenesis (from differentiated cells, DM15), the TSH-induced S.I. was lower in GO conditioned medium but not in non-GO, in correlating with the abundance of TSHRv in GO. In the experiment using M22 we noted that the S.I was lower in GO than non-GO at day 0 and after differentiation (Figure 4).

## 4. Discussion

We showed that *thyrostimulin* is unlikely to play a role in the orbit, although we could not exclude thyrostimulin production [14] by other orbital components not studied. Our results are consistent with those of Lantz et al., who also investigated orbital tissues [25]. Both orbital studies contrast with the work of Sun et al., who showed that thyrostimulin functions as an ovarian paracrine regulator [26], and also of Bassett et al., who reported the regulation of osteoblast formation in bone by thyrostimulin in early life [15]. Tissue distribution analyses showed that the α2 subunit exhibits a broader distribution than the β5 subunit; however, both transcripts were found to be expressed in human retina, pituitary, skin and testis [16,27]. Whilst it still possible that α2 can act as monomer or heteromerise with unknown ligands, current animal model data does not support this hypothesis [16,28].

Our study demonstrated that the *TSHRv* was more abundant than the full length receptor, and its expression increased during differentiation. The variant transcript is linked with the reduction of cAMP stimulation index, which suggests that *TSHR* activation was altered through interfering in TSH or TSAB binding by TSHRv. The activation of *TSHR* is clinically correlated with the severity of GO [9]. Our study presents the autoregulation of TSHR activity by TSHRv, which suggests a potential biomarker for GO. Further investigation is needed. Previous thyroid tissue northern blot analysis identified the expected full-length transcript plus two further transcripts at 1.3 and 1.6 kb [17]. We have demonstrated that the more abundant of these TSHR isoforms, 1.3kb, is also present in other locations where TSHR activation contributes to pathology, such as GO.

We confirmed our previous findings and those of others, i.e., that *TSHR* expression was very low in undifferentiated OF and increased with adipogenesis. In contrast to previous findings, we found no difference with regards to *TSHR* transcript expression between GO and non-GO [5,29]. This discrepancy can be explained by different housekeeping gene and experimental techniques used to quantify *TSHR*. We used quantitative PCR analysis in which absolute numbers of *TSHR* transcripts were reported per *APRT* housekeeping gene; this contrasts with Kumar et al., who reported relative quantification of the *TSHR* compared to the *18S RNA* [6,29]. It is well known that the use of different housekeeping genes will influence the results of gene expression. However, our western blot analysis of full length TSHR protein using 2C11 antibodies was in agreement with results published elsewhere, despite using of different techniques (i.e., flow cytometry and immunocytochemistry methods) [5,29,30].

We demonstrated that TSHR activation of undifferentiated OF (prior to the start of adipogenesis) leads to a cAMP reduction, suggesting that the receptor is coupled to Gi in these cells. Others have demonstrated different TSHR activation signalling via p70s6 kinase [31], which may result from Gq or Gs-beta/gamma signalling. To our knowledge, this is the first report of the receptor coupling to an inhibitory G protein. This finding addresses our previous failure to stimulate adipogenesis using gain-of-function mutants of the TSHR [12], and is in agreement with a study by van Ziejl et al., who investigated hyaluronan production using TSH/TSAB [32]. However, our finding contrasts with the studies of Neumann and colleagues, who demonstrated an increase in M22-mediated cAMP production, even at baseline. Of note, these authors maintained their OF in a semi-adipogenic medium, which may have contributed to increased TSHR expression [33].

The TSHRv we used in our experiments was composed of 253 amino acids, and was predicted to have five of the six N-linked glycosylation sites. Previous studies by Rapoport and colleagues showed that a TSHR truncated to 261 amino acids was unable to bind TSH [34]. The TSHR receptor contains nine leucine rich repeat (LRR) regions which have been shown to act as the binding sites for TSH/TSAB [35]. Two of the LRR are located in exon 9, but the unique sequence of 22 amino acids contributed by intron 8 are likely to be sufficient to complete the 8th LRR, and thus, to enable this variant to bind TSH/TSAB.

The TSHR variant could act in several ways: (i) As a TSHR/TSAB binding protein to neutralise their effects and inhibit TSHR activation, as suggested in the current study; (ii) A potent autoantigen which could attract T cells to the orbit and contribute to the GO pathology; (iii) Since most GD/GO patients have a mixture of TSAB and TSHR-blocking antibodies (TBABs) which bind preferentially to the N’ and C’ termini of the LRR, respectively, the variant might bind TBAB, and thus, allow TSAB to predominate.

Regulation of *TSHR* alternative splicing is generally unknown. Proximal *TSHR* promotor or intron 1 single-nucleotide polymorphisms (SNP) have been associated with increased levels of the *TSHR* variant in thyroid tissue and thymus, with a consequent impact on central tolerance mechanisms [36]. However GD patients with GO have not been shown to have a higher prevalence of disease-associated *TSHR* intronic SNP than GD patients without GO.

In summary, we showed that *thyrostimulin* does not present in orbital adipose tissue. Both *TSHR* and *TSHRv* are present in the orbit, and *TSHRv* are more abundant than full length *TSHR* in GO compared to non-GO from both in vitro and ex vivo analyses. Secreted TSHRv was detected, which may alter intracellular *TSHR* signalling through cAMP production and impact GO pathogenesis.

## Figures and Tables

**Table 1:** Primer sequences used in the experiments for the *TSHR* isoforms and *thyrostimulin* subunits.

	Accession Number	Forward	Reverse	Amplicon Size (bp)
*hAPRT*	NM_001030018.1	GCTGCGTGCTCATCCGAAAG	CCTTAAGCGAGGTCAGCTCC	247
*hLPL*	NC_000008.11	GAGATTTCTCTGTATGGACC	CTGCAAATGAGACACTTTCTC	275
*hTSHR*	NM_000369	GTGTCACTGCCCTTCCATCCA	GGGGCTATTCAAGGCATTCACAGA	254
*hTSHRv*	NM_001018036	CCTCCTAAAGTTCCTTGGCATT	AGGACTTTCTTCCAAGAGGTAG	338
*hα2*	NM_130769.3	CTCGGAAGTGATGCCTATGGC	CTAGTAGCGAGAGAGGCGAC	400
*hβ5*	AF403430	ATGAAGCTGGCATTCCTCTTC	CTGTTGGGCAGCTTGACAGTC	296

**Table 2:** cAMP response (expressed in fold changes from unstimulated samples, Stimulation Index (SI)) in OF pre (day0) and post-adipogenesis (day 15) in response to TSH 5 mU/mL and M22 0.2 ng/µL. Results are expressed as median ± interquartile range. NO, non-GO (NO); GO, Graves’ Orbitopathy.

Day of Culture	Treatment	cAMP Stimulation Index (SI)	
		NO	GO
Day 0	TSH	1.30 ± 0.11	0.29 ± 0.08 *
	M22	0.84 ± 0.03	0.75 ± 0.02 *
Day 15	TSH	3.93 ± 1.64	1.55 ± 0.45
	M22	1.89 ± 0.15	1.05 ± 0.28

* Mann-Whitney, *p*-value < 0.05 GO compared to non-GO.

## Data Availability

The data presented in this study as in Figure 1, Figure 2, Figure 3 and Figure 4, Table 1 and Table 2 and Supplementary Figures S1–S5.

## Supplementary Materials

Supplementary materials can be found at https://www.mdpi.com/article/10.3390/cimb43030126/s1. Contain five supplementary figures published online alongside this manuscript.

## Abbreviations

GO	Graves’ Orbitopathy
TSHR	Thyrotropin receptor
TSHRv	TSHR variants
OF	Orbital preadipocytes/fibroblasts
TSAB	Thyroid stimulating antibodies
GD	Graves’ disease
