# Supplementary material for: Expression of Endogenous Putative TSH Binding Protein in Orbit
Source: Curr Issues Mol Biol. 2021 Oct 27;43(3):1794–804. doi: 10.3390/cimb43030126 (PMC8928972; doi:10.3390/cimb43030126)
Supplement: Supplementary file 1 [file cimb-43-00126-s001.zip › cimb-1281320-supplementary.pdf]

current issues in molecular biology

MDPI

Supplementary Materials

Expression of Endogenous Putative TSH Binding Protein in Orbit

Mohd Shazli Draman [1,2], Fiona Grennan-Jones [1], Peter Taylor [1], Ilaria Muller [1,3,4], Sam Evans [5], Anjana Haridas [5], Daniel S. Morris [5], D. Aled Rees [1], Carol Lane [5], Colin Dayan [1], Lei Zhang [1,*,†] and Marian Ludgate [1,†]

1 School of Medicine, Cardiff University, Heath Park, Cardiff CF14 4XN, UK; shazlidraman@gmail.com (M.S.D.); grennanjonesfa@icloud.com (F.G.-J.); taylorpn@cardiff.ac.uk (P.T.); ilaria.muller@unimi.it (I.M.); ReesDA@cardiff.ac.uk (D.A.R.); DayanCM@cardiff.ac.uk (C.D.)
2 KPJ Healthcare University College, Kota Seriemas, Nilai 71800, Negeri Sembilan, Malaysia
3 Department of Clinical Sciences and Community Health, University of Milan, 20122 Milan, Italy
4 Department of Endocrinology, Fondazione IRCCS Ca' Granda Ospedale Maggiore Policlinico, 28-20122 Milan, Italy
5 Department of Ophthalmology, Cardiff & Vale University Health Board, Cardiff CF14 4XW, UK; samboevans@mac.com (S.E.); anjana@doctors.org.uk (A.H.); dsm@doctors.org.uk (D.M.); carollanedm@gmail.com (C.L.)
* Correspondence: zhangL14@cf.ac.uk; Tel.: +44-292-074-2343; Fax: +44-292-0744-671
† These authors joint senior author.

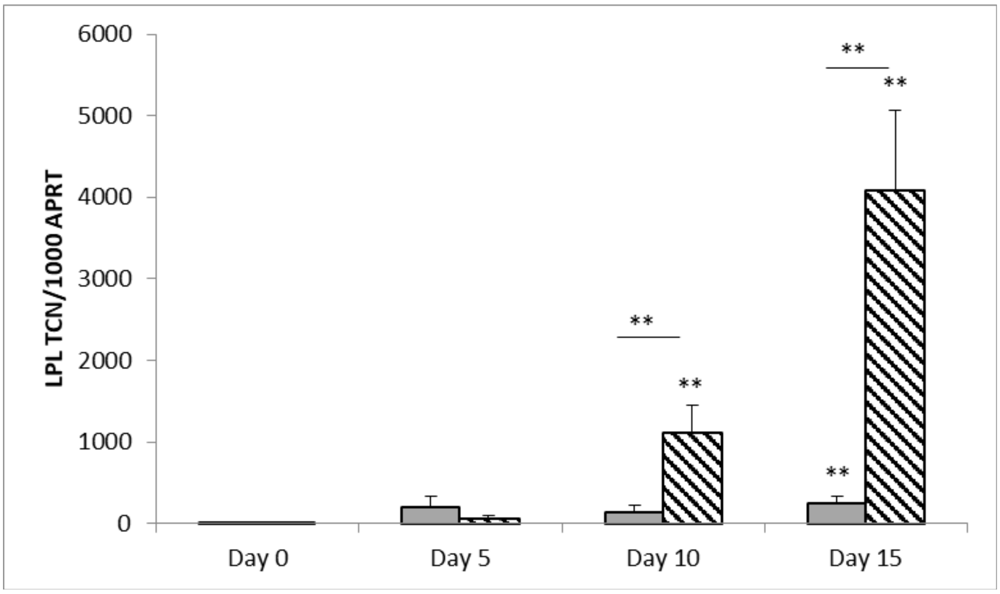

Supplementary Figure S1. Marker of adipogenesis Lipoprotein lipase (*LPL*) transcripts were measured in orbital preadipocyte-fibroblasts (OF) from patients with Graves' orbitopathy (GO) (Stippled bar, *n* = 5) and non-GO (grey bar, *n* = 5) at various time points at baseline (Day 0) and during adipogenesis (Day 5, 10, 15). Results are presented as LPLtranscript copy number per 1000 copies of the housekeeper *APRT*. Results are expressed as mean ± SEM; ** *p*-value < 0.01 compared to day 0 on each respective day; horizontal bar represent comparison between non-GO and GO.

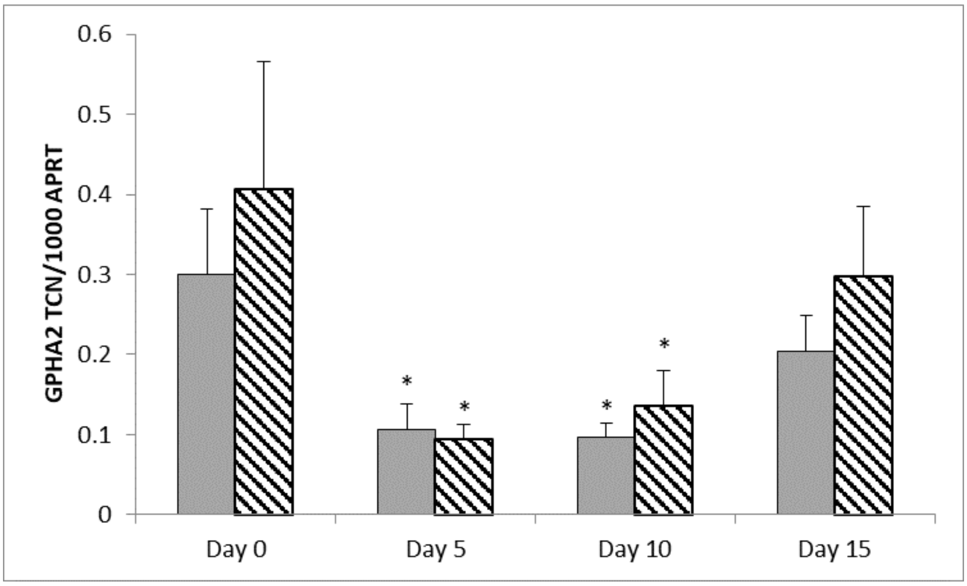

Supplementary Figure S2. Transcripts for the *thyrostimulin α2* subunit were measured in OF from patients with GO (Stippled bar, n = 5) and non-GO (grey bar, n = 5) at various time points before (Day 0) and during adipogenesis (Day 5, 10, 15). Results are expressed as transcript copy number of α2 per 1000 copies of the housekeeper *APRT*. Results are presented as mean ± SEM; * p-value < 0.05 for days 5, 10, 15 compared to equivalent day 0 for GO or non-GO.

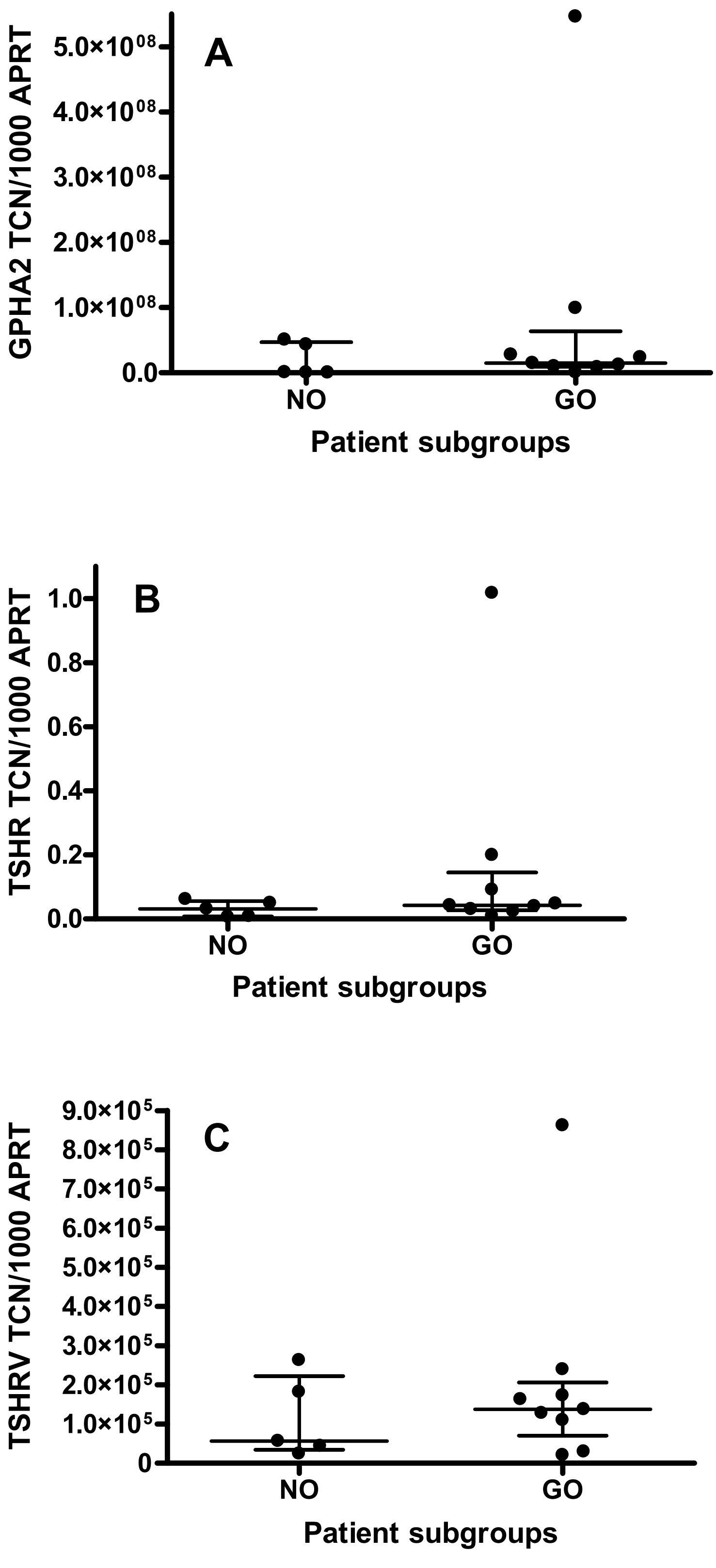

Supplementary Figure S3. Ex vivo analysis of orbital adipose tissue from non-GO individuals (NO, n = 5) and patients with GO (n = 9) for transcripts of A, *thyrostimulin α2* subunit; B, *TSHR*; C, the *TSHRv*. Results are expressed as transcript copy number per 1000 copies of the housekeeper *APRT*. Results are presented as median ± interquartile range.

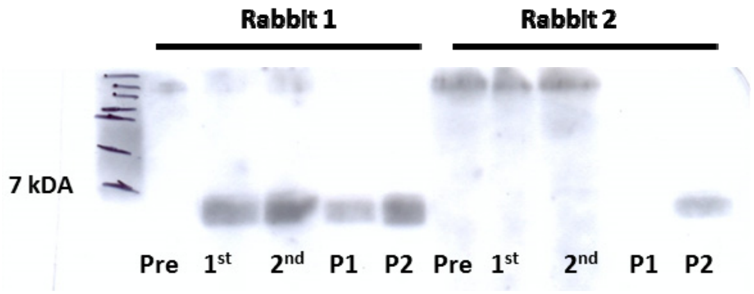

Supplementary Figure S4. Successful generation of antibody to unique TSHRv peptide.

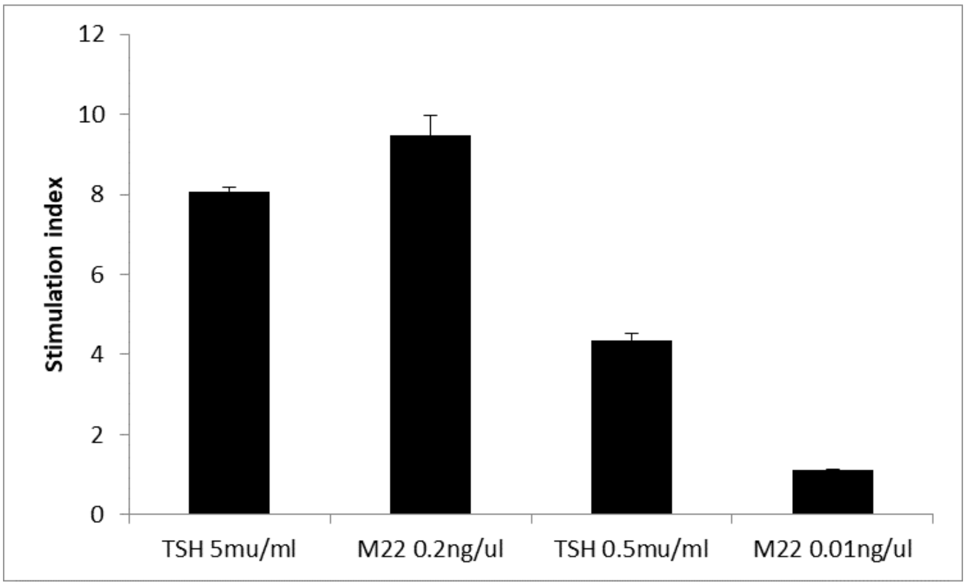

Supplementary Figure S5. Luminescent bioassay to measure cAMP following TSHR activation. Stimulation index in the presence of varying doses of TSH and monoclonal TSAB M22.
